# A Smartwatch System for Continuous Monitoring of Atrial Fibrillation in Older Adults After Stroke or Transient Ischemic Attack: Application Design Study

**DOI:** 10.2196/41691

**Published:** 2023-02-13

**Authors:** Dong Han, Eric Y Ding, Chaeho Cho, Haewook Jung, Emily L Dickson, Fahimeh Mohagheghian, Andrew G Peitzsch, Danielle DiMezza, Khanh-Van Tran, David D McManus, Ki H Chon

**Affiliations:** 1 Department of Biomedical Engineering University of Connecticut Storrs, CT United States; 2 Department of Medicine University of Massachusetts Chan Medical School Worcester, MA United States; 3 Zebra Technologies Inc Holtsville, NY United States; 4 SSP Seoul Republic of Korea; 5 Mediporte Co, Ltd Gyeonggi-do Republic of Korea; 6 College of Osteopathic Medicine Des Moines University Des Moines, IA United States; 7 iSpecimen Inc Lexington, MA United States

**Keywords:** atrial fibrillation, stroke, smartwatch app, smartphone apps, wearable devices, user experience, older adults, mobile phone

## Abstract

**Background:**

The prevalence of atrial fibrillation (AF) increases with age and can lead to stroke. Therefore, older adults may benefit the most from AF screening. However, older adult populations tend to lag more than younger groups in the adoption of, and comfort with, the use of mobile health (mHealth) apps. Furthermore, although mobile apps that can detect AF are available to the public, most are designed for intermittent AF detection and for younger users. No app designed for long-term AF monitoring has released detailed system design specifications that can handle large data collections, especially in this age group.

**Objective:**

This study aimed to design an innovative smartwatch-based AF monitoring mHealth solution in collaboration with older adult participants and clinicians.

**Methods:**

The Pulsewatch system is designed to link smartwatches and smartphone apps, a website for data verification, and user data organization on a cloud server. The smartwatch in the Pulsewatch system is designed to continuously monitor the pulse rate with embedded AF detection algorithms, and the smartphone in the Pulsewatch system is designed to serve as the data-transferring hub to the cloud storage server.

**Results:**

We implemented the Pulsewatch system based on the functionality that patients and caregivers recommended. The user interfaces of the smartwatch and smartphone apps were specifically designed for older adults at risk for AF. We improved our Pulsewatch system based on feedback from focus groups consisting of patients with stroke and clinicians. The Pulsewatch system was used by the intervention group for up to 6 weeks in the 2 phases of our randomized clinical trial. At the conclusion of phase 1, 90 trial participants who had used the Pulsewatch app and smartwatch for 14 days completed a System Usability Scale to assess the usability of the Pulsewatch system; of 88 participants, 56 (64%) endorsed that the smartwatch app is “easy to use.” For phases 1 and 2 of the study, we collected 9224.4 hours of smartwatch recordings from the participants. The longest recording streak in phase 2 was 21 days of consecutive recordings out of the 30 days of data collection.

**Conclusions:**

This is one of the first studies to provide a detailed design for a smartphone-smartwatch dyad for ambulatory AF monitoring. In this paper, we report on the system’s usability and opportunities to increase the acceptability of mHealth solutions among older patients with cognitive impairment.

**Trial Registration:**

ClinicalTrials.gov NCT03761394; https://www.clinicaltrials.gov/ct2/show/NCT03761394

**International Registered Report Identifier (IRRID):**

RR2-10.1016/j.cvdhj.2021.07.002

## Introduction

### Background

Atrial fibrillation (AF) is the most frequent cardiac arrhythmia [1], and the prevalence of AF in the United States is increasing, from an estimated 5.2 million in 2010 to a projected 12.1 million in 2030 [2]. AF, whether paroxysmal, persistent, or permanent, and whether symptomatic or silent, significantly increases the risk of thromboembolic ischemic stroke [3]. Owing to the difficulty in diagnosis, paroxysmal AF (pAF) is the most common AF pattern found in all patients presenting with acute ischemic stroke [4]. Diagnosing pAF remains critically important and often requires monitoring for >24 hours [5]. The popularity of noninvasive wearable devices and user-friendly, informative mobile apps may play an important role in long-term heart rhythm monitoring for the population at high risk of pAF [6]. In addition, the COVID-19 pandemic has fundamentally altered the landscape of clinical care in the United States, with many older adults and their clinicians shifting to internet-based visits and increasing the acceptability of wearable devices as medical monitors. Because the prevalence of AF increases with age, reaching 9% in people ≥80 years [7], the ideal population to screen for AF is of older adults. However, familiarity with wearable devices remains low among older adults. Physical, as well as cognitive, impairments can interfere with the ability of older adults to use mobile health (mHealth) apps and commercial wearables [8]. We aimed to design a system for AF monitoring that would be highly usable by older adults by incorporating their feedback into the design of a smartphone app.

Traditional electrocardiogram (ECG) monitoring uses hydrogel-based adhesive electrodes, which leads to poor patient acceptance and usability in long-term monitoring apps. Most current mobile noninvasive technologies with automated pulse or ECG acquisition have highly accurate AF detection, calculated from embedded advanced signal processing algorithms [6]. However, initial manifestations of this technically required an individual to perform self-checks by placing fingers on a smartphone camera lens or pairing them with an ECG unit for 30 seconds to 2 minutes of recording. This *spot-check* approach does not provide the continuous, passive monitoring needed to detect brief episodes of AF in high-risk populations, such as those with cryptogenic stroke.

### Prior Work

A better method to monitor pAF passively and near-continuously is to use a photoplethysmography (PPG) sensor on the back of a smartwatch to record pulse information. Recently, Apple Heart study [9,10], Huawei Heart study [11,12], and Fitbit Heart study [13] evaluated systems that use this approach to monitor pAF. However, all 3 studies targeted users who already owned each brand’s smartphones and smartwatches. Therefore, the Apple Heart study, Huawei Heart study, and Fitbit Heart study were skewed toward younger participants. In fact, each study included only 5.9%, 1.8%, and 12.5% of the total cohort of participants aged ≥65 years, respectively. No details were provided on whether the apps were specifically designed by or for older adults, and no details on the design of the rhythm collection system have been described [10-13]. In addition, Including the details of the design is important, as long-term monitoring that generates enormous data could overload wearable devices that have limited storage.

### Goal of This Study

As with any novel technological development, end user guidance in design and development is paramount. This is especially true for pAF monitoring because the target population of older stroke patients is unique, slower to adopt new technology, and understudied. Accordingly, in our clinical trial [14], we collaborated with survivors of stroke and their clinicians to develop Pulsewatch, an innovative smartwatch-based AF detection mHealth system. This comprised a Samsung smartwatch for long-term (up to 6 weeks), near-continuous (24 h/d) pulse monitoring and on-demand ECG into which we embedded our novel algorithms for noise elimination, contact sensing, and automated AF detection and which communicated with a smartphone app with user interface (UI). In this study, we provide the details of our system design and its acceptability among older survivors of stroke or transient ischemic attack (TIA).

## Methods

In this section, we discuss the design of the functionalities of the Pulsewatch system. Details of the final implementation of the Pulsewatch system are provided in the *Results* section.

### Overview of the Functionality of the Pulsewatch System

The Pulsewatch system consisted of a pair of smartphone and smartwatch apps and was intended to be used for at least 6 weeks in an at-home ambulatory setting by older adults who survived a stroke or TIA [14]. The aim of the Pulsewatch system was to provide a passive AF monitoring solution with minimal user attention required during recording with real-time display of the monitoring results. Importantly, owing to the cognitive impairment of our target population, pulse monitoring had to be passive, wherein the participants were only asked to hold still when a rhythm abnormality was detected. Although the system required no action on the part of the user, users can access historical data through the Pulsewatch app. Users can input symptoms or notes for their clinicians, as the system was designed to facilitate the sharing of information about the AF status between the user and their clinicians. The participants’ clinicians can also remotely check the Pulsewatch system’s wearing time information, user symptoms, and AF detection results.

The Pulsewatch system was designed to be used by 3 groups of users, as described in Textbox 1.

During the design process of the Pulsewatch app, inputs from both patients and their clinicians were taken to facilitate communication. Our patients’ focus group consisted of 5 screened patients with a history of stroke or TIA at the University of Massachusetts Chan Medical School’s (UMass Chan) Stroke Prevention Clinic, whereas our clinicians’ focus group participants consisted of 5 clinicians (neurologists and cardiologists) from the UMass Chan Neurology and Cardiology groups. Developers (algorithm developer and software engineer) also joined the hack-a-thon and agile programming team meeting at UMass Chan to finalize the Pulsewatch system to be deployed in the clinical trial.

**Figure 1:**
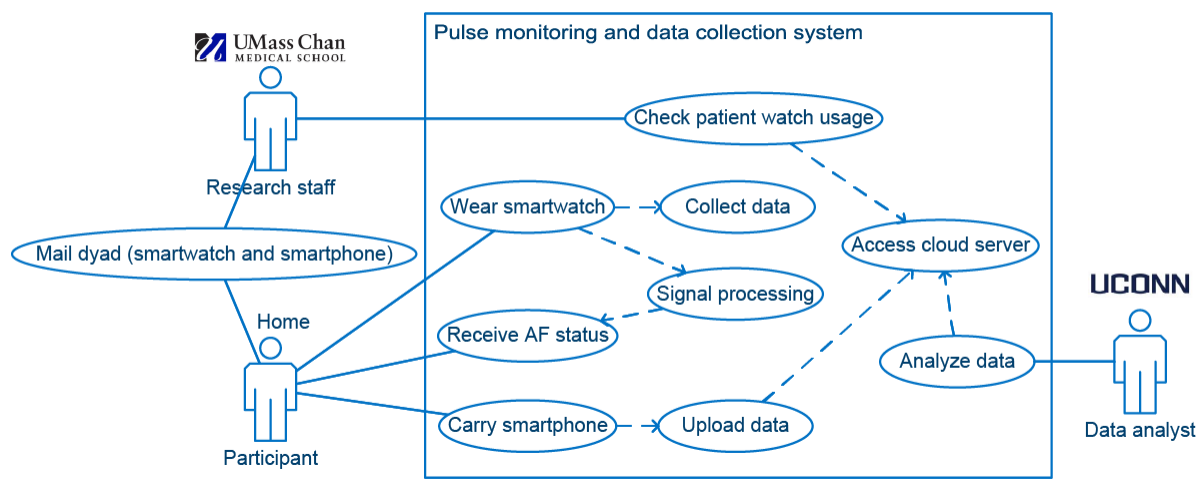
Use case diagram of the data collection system. AF: atrial fibrillation; UConn: University of Connecticut; UMass Chan: University of Massachusetts Chan Medical School.

The use case diagram [15] depicted in Figure 1 shows the main functionalities provided by our Pulsewatch system. The Pulsewatch experiment began with a smartwatch app that could automatically and independently detect AF. After a participant received the dyad composed of the smartwatch and smartphone, turned on the devices, and donned the smartwatch, the data collection and signal processing procedure automatically started without any configuration needed from the participant. We believe that this is the most feasible way to start monitoring AF in older adult participants.

**The 3 user groups for the Pulsewatch system .**

1. Participants (end users): Participants were identified using electronic health records based on diagnostic codes and then approached by our research staff at the time of a clinic visit for potential enrollment. Eligible participants had to be aged ≥50 years with a history of stroke or transient ischemic attack within the past decade. A detailed description of the target user, sample size, hypothesis of the study, and inclusion and exclusion criteria was previously described by Dickson et al [14]. If participants were interested, they provided informed consent to participate in the study and were subsequently randomized to determine whether they had received the Pulsewatch system. The study staff provided the smartwatch and smartphone devices, extensive training, and written information detailing device operation. Participants could only use the smartwatch and the smartphone assigned to them for the study.
2. Research staff (the web admin): After the initial training, the study staff provided technical and operational support over the phone when required by patients. In addition, the study staff used the user interface of the Pulsewatch web system to examine the pulse data collected from the enrolled participants (ie, they accessed the data tracking websites that we described in the *Results* section and implemented data tracking website on the cloud server), to check the secured cloud server and to provide on-time feedback for the phase 1 experiment.
3. Data analysts (the local administrator): Data analysts from the University of Connecticut had access to the entire Pulsewatch system, including the secured cloud server for administrative management and future data processing.

### Ethics Approval

Formal ethical approval for this study was obtained from the Institutional Review Board of the UMass Chan’s Institutional Review Board (Approval Number H00016067). Written informed consent was obtained from all patients involved in the hack-a-thon and clinical trials. Verbal consent was obtained from clinicians enrolled in the hack-a-thon development phase. Because of the unprecedented challenges posed by COVID-19 regarding in-person human participant research, we adopted an alternate protocol to allow for all study encounters to occur over the phone [14]. The consent form was adopted for telephone and approved by the Institutional Review, and eligible participants were contacted via their contact information in the electronic health record and consented [14]. This alternate remote protocol was initiated in July 2020, and all participants who had been approached over the phone were also offered the option to participate in person as per the original study protocol [14].

All participants’ identifiable information was deidentified by the UMass team. The authors designed the study and gathered and analyzed the data according to the Helsinki Declaration guidelines for human research.

The 5 patient participants who completed the hack-a-thon were each awarded a US $50 visa gift card. During the clinical trial, enrolled participants received a US $100 visa gift card after completing the baseline questionnaire for phase 1 of the study and another US $100 gift card for completing the follow-up questionnaire. Participants who were randomly selected for a usability interview received an additional US $60 visa gift card. After completing phase 2 and the 44- day follow-up questionnaire, the participants were compensated with a US $50 visa gift card.

### Functionality of Independent AF Detection on Smartwatch

**Figure 2:**
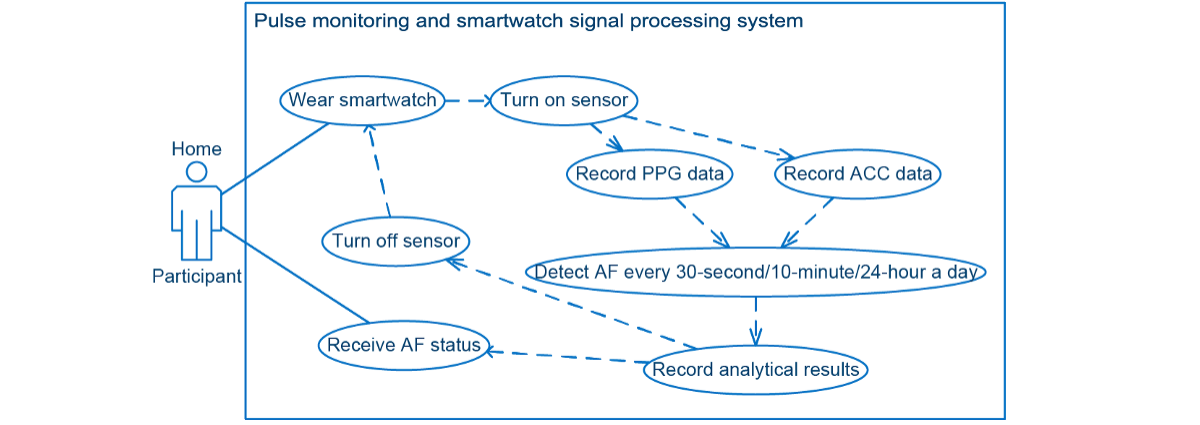
Use case diagram of pulse monitoring (smartwatch signal processing) system. ACC: accelerometry; AF: atrial fibrillation; PPG: photoplethysmography.

Figure 2 illustrates the core functionality and highlights of the Pulsewatch system, that is, the signal processing system on the smartwatch.

We designed a novel sensor modulation program to turn the sensor on every 10 minutes to preserve the smartwatch battery while maximizing the monitoring span. The duration of the sensor-on stage was 5 minutes but could be extended based on the instantaneous AF detection results [14].

### Functionality of Automated File Transferring Between Smartwatch and Smartphone

**Figure 3:**
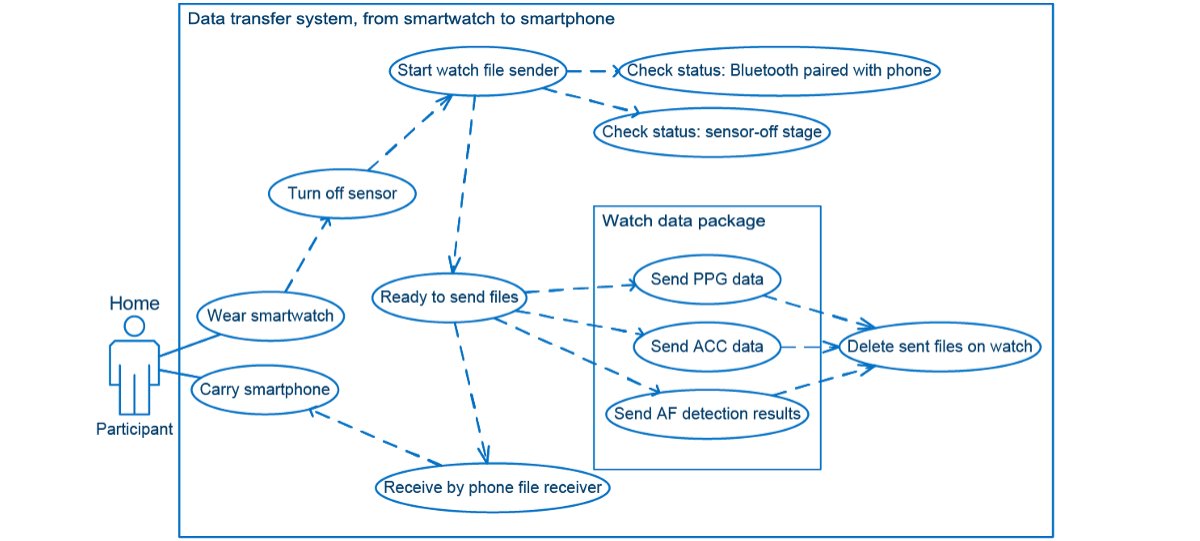
Use case diagram for phone-watch file transfer system. ACC: accelerometry; AF: atrial fibrillation; PPG: photoplethysmography.

Once data were collected and processed on the smartwatch, the next critical step was when and how to upload them to the smartphone so that the storage on the watch could be freed. As shown in Figure 3, the file transferring procedure initiated by the watch occurred immediately when the sensor was turned off, thereby ensuring that no new files were created. The file sender program inside the smartwatch app first confirmed that a Bluetooth connection was established between the smartwatch and the paired smartphone. When the criteria were met, a file transfer process was initiated. Three types of data were transferred from the watch to the phone: PPG raw data, accelerometry (ACC) raw data, and results of signal processing and AF detection methods. Details of the output data are provided in the *Results* section. Once the smartphone successfully received the watch data, the watch app deleted the transferred data to free the storage space for future data collection. The smartphone app file receiver application programming interface (API) continuously ran in the background of the smartphone to ensure that any spontaneous file uploading requests were received from the watch.

### Functionality of Automated File Backup Between the Smartphone and the Cloud Server

**Figure 4:**
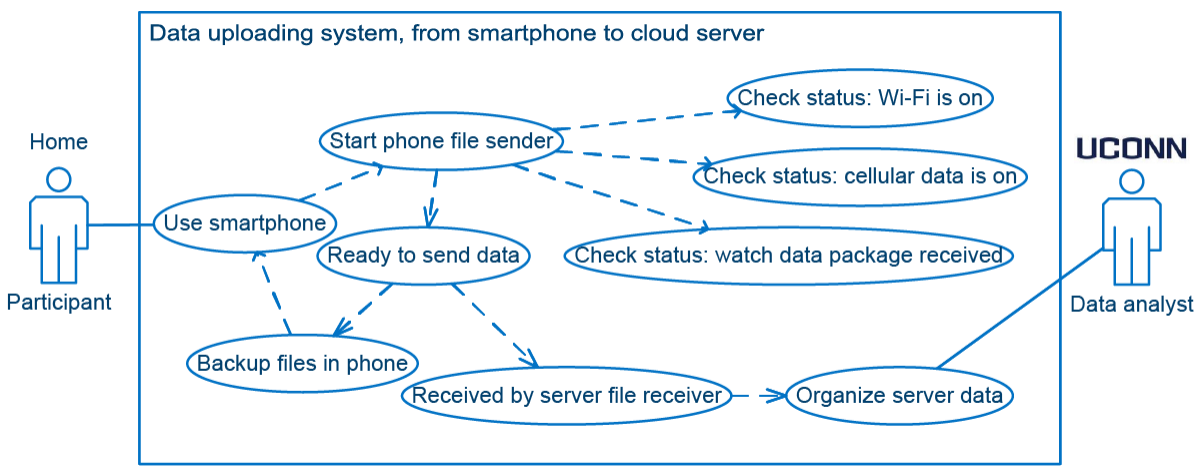
Use case diagram of the phone-server file transfer system. UConn: University of Connecticut.

Even if the smartphone received data transferred from the smartwatch, the physical dyad could be damaged or lost at any moment. To address this, we designed a Pulsewatch system to ensure that the data were backed up to the cloud server. As shown in Figure 4, when the smartphone was turned on, the file sender in the Pulsewatch phone app automatically ran in the background constantly to ensure that files were uploaded to the cloud at any given moment. Before uploading unsent files to the server, the file sender must establish internet connection, either through Wi-Fi or cellular data. Once a file was uploaded through the internet connection successfully, the phone app did not delete it but instead moved it to another folder to have a second local backup. This was because during transfer, the data could be corrupted, and therefore, it was critical to keep the original files in the phone.

### Functionality of Symptom Logging in the Smartphone

**Figure 5:**
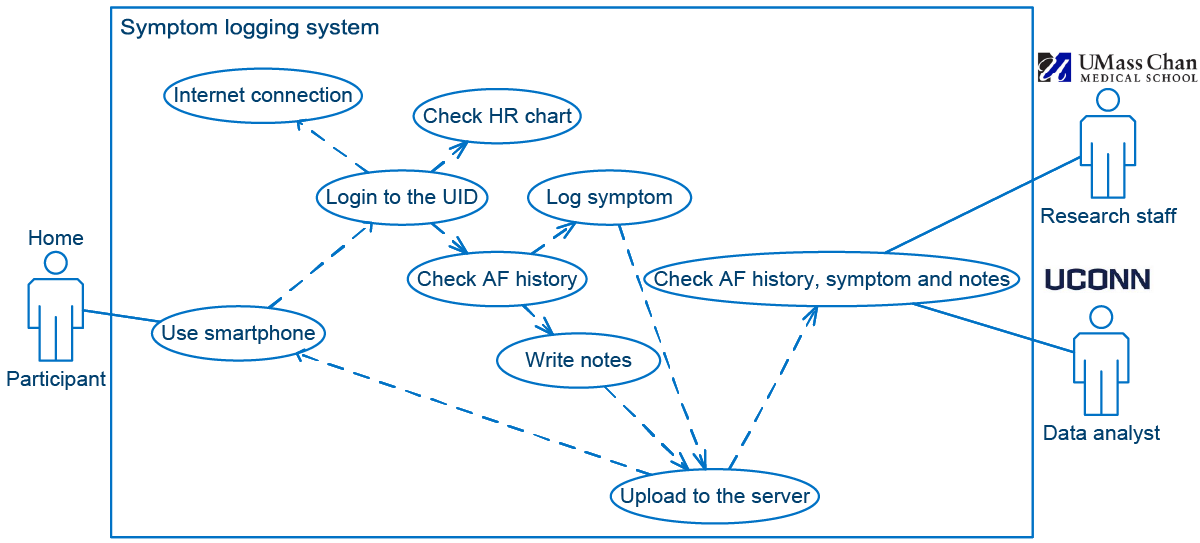
Use case diagram of the symptom logging system. AF: atrial fibrillation; HR: heart rate; UConn: University of Connecticut; UID: user ID; UMass Chan: University of Massachusetts Chan Medical School.

An important functionality required by cardiologists, as shown in Figure 5, was participants’ symptom tracking within the Pulsewatch smartphone app, which mirrors the same functionality as clinical heart rhythm monitors. Regardless of the AF status results in the phone app, participants were able to select predefined symptoms or log free text in the smartphone app at any time to inform their clinician. To separate different participants’ inputs on the cloud, the participants had to first log-in to the phone app with their user ID (UID) and password. This log-in process required internet connection to contact the cloud server for log-in credentials. After log-in, the participants used the start time point from when the watch sensor was turned on every 10 minutes to input their symptoms and notes. Once the participant clicked the save button, all edits were automatically uploaded to the server through an internet connection. The UMass research staff could read the participant’s symptom input immediately on the webpage.

## Results

In this section, we explain the implementation of all the functionalities proposed in the *Methods* section for our Pulsewatch system. We then describe the output of our Pulsewatch clinical trial, including the total number of recordings collected from the Pulsewatch system and the usability ratings of our system among our participants.

### Implemented Structure of the Pulsewatch System

**Figure 6:**
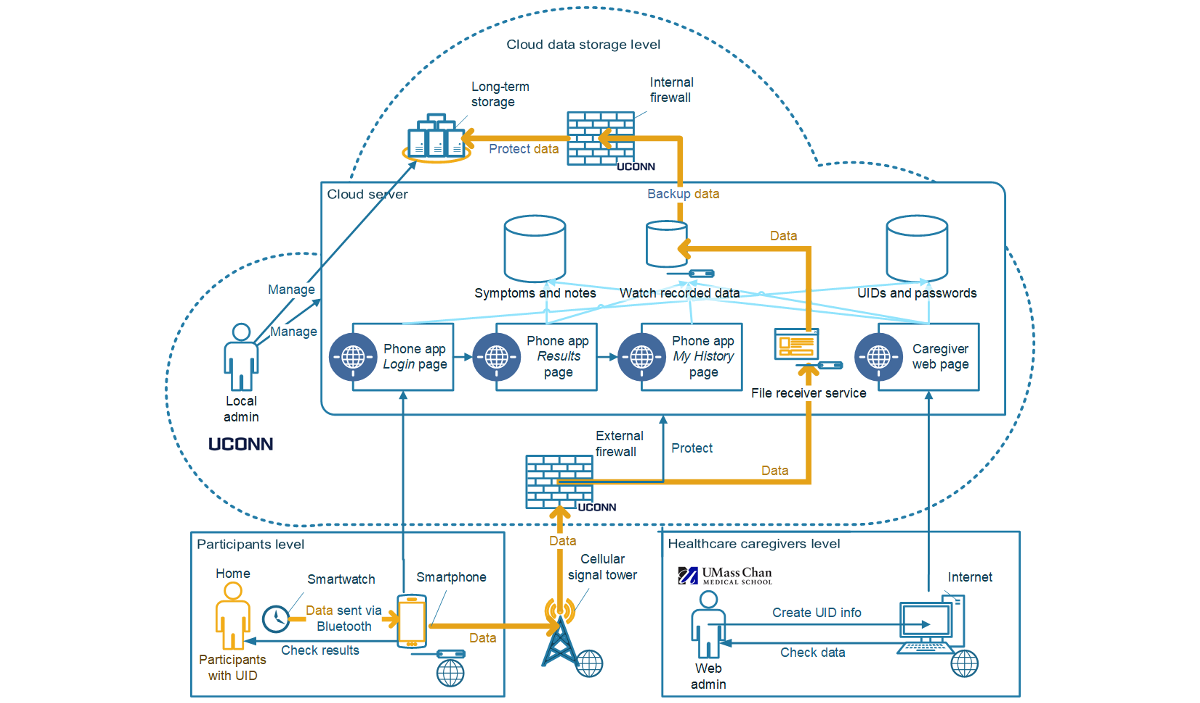
Structure of the Pulsewatch system. Admin: administrator; UConn: University of Connecticut; UID: user ID; UMass Chan: University of Massachusetts Chan Medical School.

On the basis of the functionality mentioned in the *Overview of the Functionality of the Pulsewatch System* in the *Methods* section, we designed the structure of our Pulsewatch system, as shown in Figure 6.

At near real-time speed (<1 s), the smartwatch processed the recorded data and displayed an AF status (normal or abnormal) on the watch UI, together with the time of day. There was no notification of AF status other than the watch face color and text, reflecting the preferences of older adult patients. After collecting and processing the data, they were transferred to the paired smartphone via self-initiated communication within the dyad. The data were backed up both in the phone’s local storage and on the cloud server through the phone app.

### Implemented UI of the Smartwatch App

**Figure 7:**
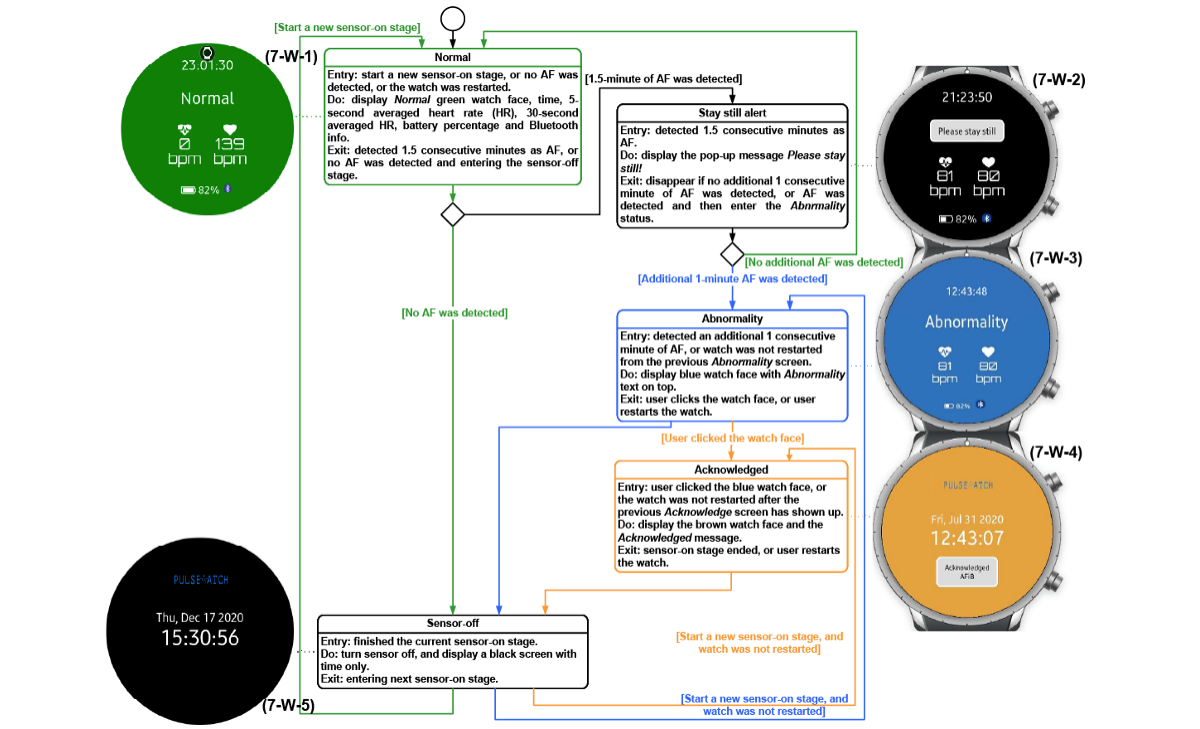
The state machine of the Pulsewatch smartwatch app. AF: atrial fibrillation; bpm: beats per minute; HR: heart rate.

On the basis of the functionality requested in Figure 2, we implemented the logic as shown in Figure 7. During the sensor-on stage, PPG and ACC data were recorded, and AF detection was performed [16,17] based on the PPG heart rate value [18] from clean PPG segments [16]. The results of AF detection were displayed accordingly on the watch face. The enrolled participants were able to observe the AF status from the smartwatch face passively when they checked the time displayed on the watch. If the system detected AF, the status lingered on the watch face until the participant clicked the watch face to acknowledge it. This AF detection cycle was repeated all day while the watch was powered on.

For the watch app UI shown in Figure 7, the AF status was displayed with the watch face color and text at the top, based on suggestions from the patient focus group members in the hack-a-thon [14]. To avoid inducing worry among participants, the watch face color for abnormality was deliberately chosen as blue (in lieu of a red color). Color blindness was considered and factored into the choice of warning color, following a suggestion by clinician participants in the hack-a-thon [14]. In addition to the AF status (normal, possible AF active, possible AF previously detected), the watch face also displays the time and 2 distinct heart rate features. Details of the smartwatch UI are provided in Multimedia Appendix 1. As the sensor was turned off half of the time, we used the darkest color for the background to minimize the brightness of the watch face and disturbance to participants’ sleep. The frequency of *normal* watch face could be high as well, if the participant did not have long episodes of AF or had lots of motion artifact; thus, we used a darker green color in (4-W-1) of Figure 7, compared with the dazzling green color on the watch face shown in the *normal* screen of Figure 3 in [14]. We did not design any night mode for the watch face in case participants preferred to be woken up at night for any AF alert. To indicate the Bluetooth connection and the remaining battery percentage, we added 2 icons at the bottom of the watch face to help participants debug the file transferring issue if the study staff had not seen their data for several days in phase I.

### Implementation of Smartwatch App

**Figure 8:**
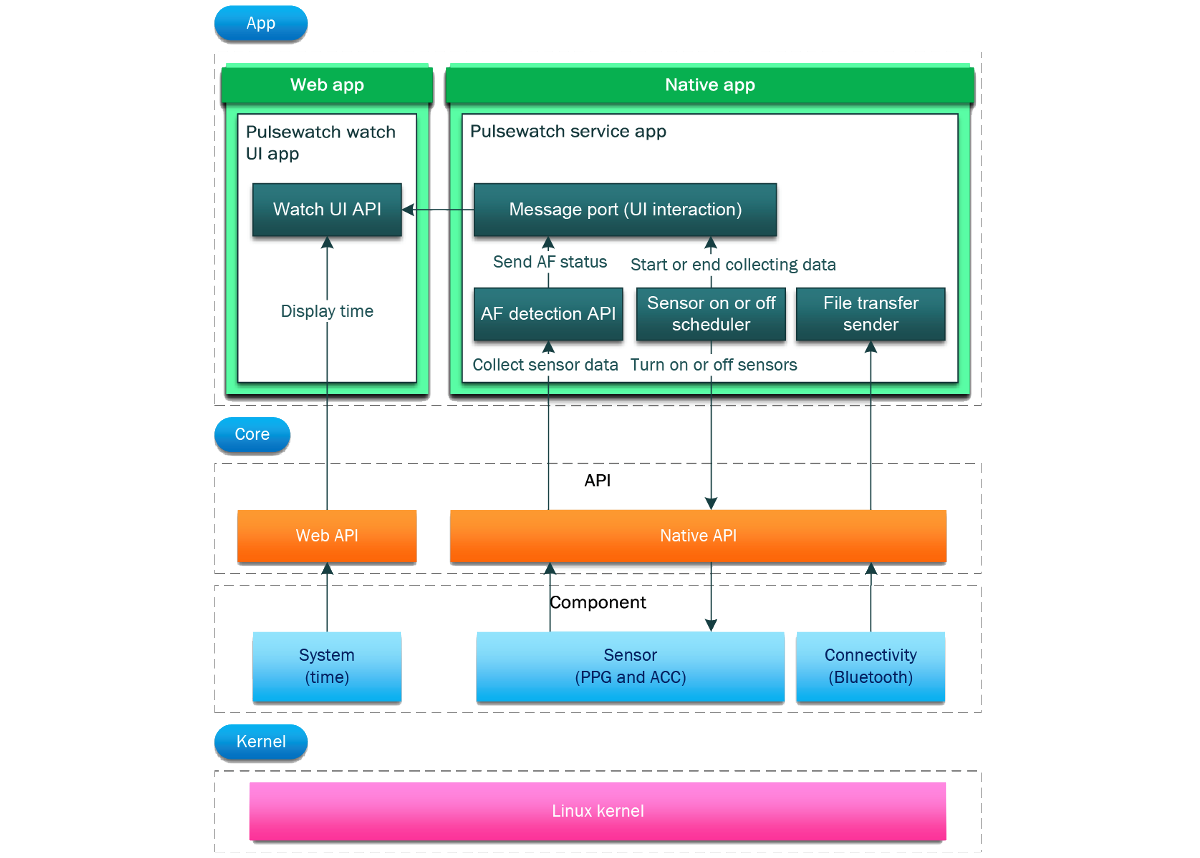
The architecture of the Pulsewatch smartwatch app. ACC: accelerometry; AF: atrial fibrillation; API: application programming interface; PPG: photoplethysmography; UI: user interface.

Before we introduced the final implementation of the watch app, we needed to provide background knowledge on the paradigms of mobile watch apps. On the basis of the required functionalities, we designed the watch app architecture shown in Figure 8 [19]. The Pulsewatch watch application uses both types of apps that the Samsung Tizen (Samsung) Wearable OS supports: a web app, which is for the watch face application, and a native application, which is for the Pulsewatch service application—a job manager for calling the sensors to collect data or calling the algorithm to process the data. The watch face application, written in web language (ie, HTML, Cascading Style Sheets, and JavaScript), is a special type of application that runs automatically and consistently on the home screen of a watch with the Samsung Tizen Wearable OS when the watch is turned on. The watch faces shown in Figure 7 are the only apps that the participants could see. All automated procedures are described below for both native and service apps.

According to the designed functionality of the watch app (signal processing functionality in Figure 2, UI changes in Figure 7, and file transferring functionality in Figure 3), we summarize the workflow of our watch app as follows. As mentioned previously, every time the smartwatch was powered on, the watch face application ran first, followed by the native application. The watch face color or pattern changes according to the input sent from our service application through the message port. Our service application then initialized all APIs equipped and started the AF detection procedure. A service application turns on the watch sensors through the sensor scheduler and then sends the initiated sensor status to the watch face application through the same message port. Then, the AF detection API collects and processes all sensor data and transmits the AF detection results to the watch face application through the same message port. Finally, when the 5-minute sensor-on stage is completed, the sensor scheduler turns off the sensor and updates the sensor status on the watch face application through the same message port.

Every smartwatch model was powerful enough to process the pulse signal and detect AF in near real time. The initial model of the smartwatch was Gear S3 Classic (2016). After nearly a year of heavy use in the clinical trial, the battery health of the Gear S3s degraded significantly and could not support more than 3 hours of use, and this older model was discontinued by the manufacturer. We replaced it with a Galaxy Watch 3 (41 mm; 2020) watch, which had better sensors for data collection and larger RAM and internal storage compared with Gear S3. Both Gear S3 and Galaxy Watch 3 run an open source Samsung Tizen Wearable operating system (OS) from the Linux Foundation [20], allowing researchers to have access to the original sensor data using a free open-source API without any commercial license fees to Samsung [21].

### Implementation of Smartphone App

When deploying a mobile phone app, which is slightly different from the deployment of the mobile watch app, we decided to use a hybrid application [22], where we display content using the web application and then fit it inside the native application structure. The reasons for this are presented in Table 1.

**Figure 9:**
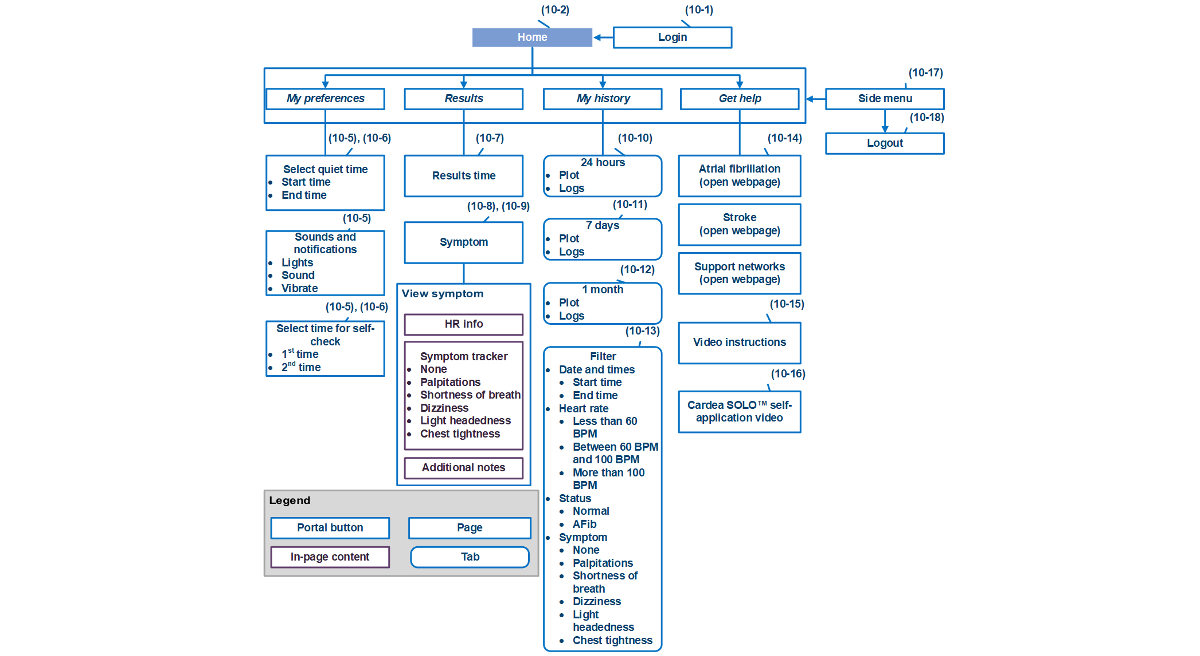
The information architecture of the Pulsewatch smartphone app. AFib: atrial fibrillation; HR: heart rate; UID: user ID.

**Figure 10:**
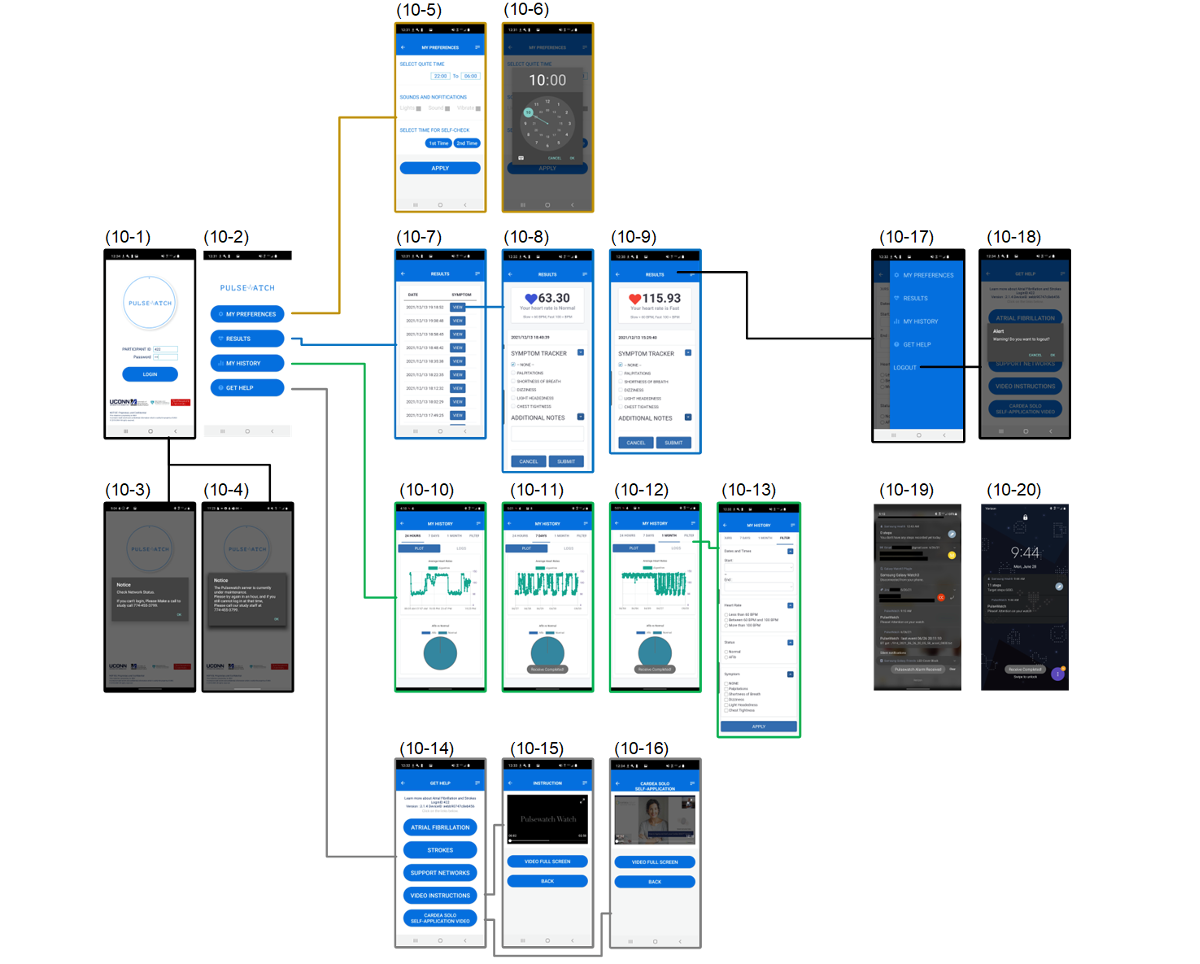
The final implemented web-based phone app user interface.

We structured the relationships between pages and other content components [23] of the Pulsewatch phone app in a top-down informational architecture, as shown in Figure 9. After log-in, the user entered the home page, which consisted of 4 large buttons for 4 main categories: “My Preferences,” “Results,” “My History,” and “Get Help.” These 4 main pages can also be navigated quickly using the side menu (screenshots 10-17 in Figure 10). Details of the content displayed on each page are provided in the Multimedia Appendix 1.

The “Login,” “Results,” and “My History” pages are actually 3 web-based apps that run on the cloud server. We chose this application type mainly because the large amount and importance of the content displayed on these 3 pages led to many UI revisions. Thus, compared with using a native app paradigm, using web apps significantly reduced the developer’s burden and end user’s pain in reinstalling the apps. However, if the cloud server was shut down for maintenance, no users could use the phone app. Therefore, it was necessary to schedule a time slot for server maintenance (eg, around 3 AM to 5 AM), because not many users were logged in and used the app during these hours.

The design of the “Login” page can shorten the dyad cleaning time (eg, removal of patient information and data) between any 2 participants in the experiment for UMass study staff. When the app was first used by a participant, the phone app required a 1-time log-in for the participants to input their UID and passwords. This process could also be performed by the study staff to reduce app use difficulties before handing out the dyad. All the data recorded from the smartwatch will be tagged with this UID. Once the experiment was finished, the UMass study coordinators were able to manually log out the current participant in the side menu from any page to prepare the dyad for the next participant. Once the current UID was logged out, the next user could not see any history of information from previous users, and the newly recorded data can be isolated from the previous data with a different UID.

**Table 1:** Decision of using hybrid application paradigm.

Factors	Native apps	Web apps	Requirements
Hardware features	Have all the access.	No access.	Bluetooth connection with smartwatch.
System features	Have all the access.	No access.	Push notifications and run smartphone app on the background.
UI^a^ Style	Match seamlessly with the system UI.	May looks less *authentic* to users.	No strict requirement on unity with system UI.
Publishing of app	Publish on app stores such as Google Play and Samsung Galaxy app store or rely on manual installation.	In-browser web page, no installation needed.	Rely on manual installation and cannot use App Store because of study confidentiality.
App updates	Must perform reinstallation.	Remotely update the content on web page, no need for reinstallation.	As few reinstallations as possible.
Internet connection	No internet required if data were all stored locally on the phone. Content display speed is fast.	Must have internet connection. Content display speed relies on internet speed.	Should not work in offline mode, must ask user to use internet so data can be backup on the cloud.
Developing time	Lengthy as more code libraries are involved.	Fast.	As fast as possible.
Cost	High.	Low.	As low as possible.

^a^UI: user interface.

The “My Preferences” and “Get Help” pages are native app pages because they need access to system-level features and have to work offline. The “My Preferences” page was mainly designed to let a user set up twice daily self-check alarms. This allows users to set the time and notification types for self-check alarms. The number of acceptable prompts that Pulsewatch can deliver per day was determined by the patient focus group participants in the hack-a-thon [14]. In addition to alarm time, users could also set a do-not-disturb time to avoid unwanted interruptions from the Pulsewatch app. Notification options such as flashlights, sound, and vibration could also be turned on or off. Self-check alarms appeared in the notification center to allow the user to tap it and open our Pulsewatch phone app quickly. This alarm could be deleted using either a swipe left or right action.

For the phone UI shown in Figure 10, a larger font size and capitalized letters were appreciated by end users [14], many of whom had visual impairments. In the phone app, a pie chart on the results page was added, as one of the participating clinicians suggested [14] that it would be important for clinicians to have a report that showed the AF burden recorded by the system.

As some active participants might wonder if the watch files were successfully uploaded to the phone, we placed a steady notification banner, using the native app structure, in the notification center to show the last time that a file was transferred from the watch to the phone. This notification banner was not visible unless the participants swiped down the notification center.

### Implemented Data Tracking Website on the Cloud Server

**Figure 11:**
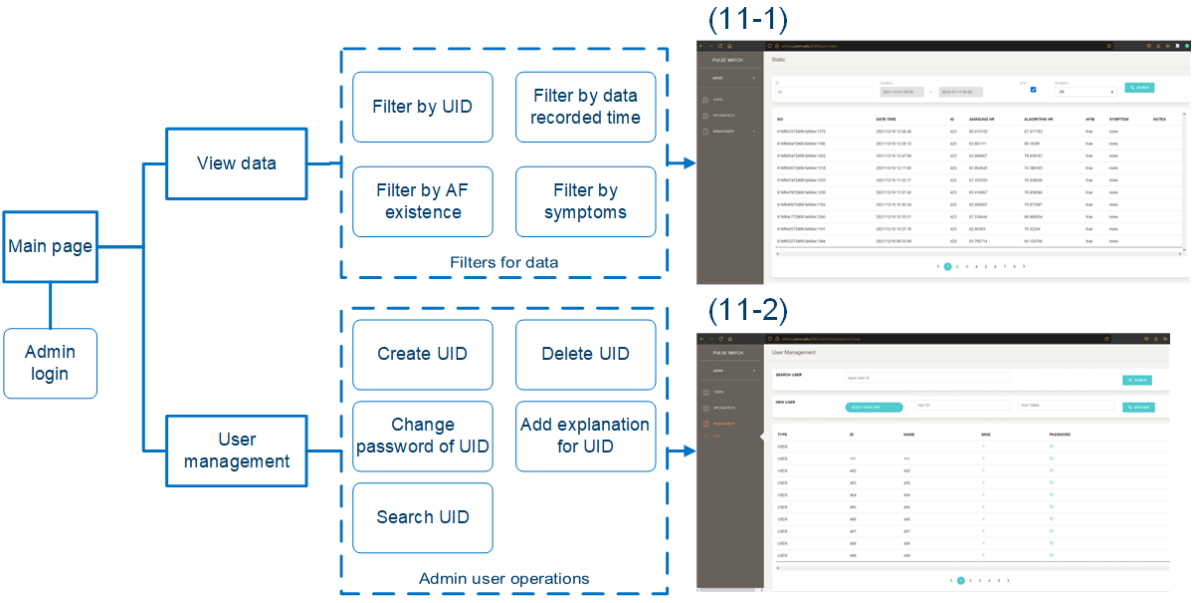
The information architecture of the data tracking website and the final implementation of the data tracking website. AF: atrial fibrillation; UID: user ID.

Most electronic health records are now web and client-server–based and use relational databases [24]. Although not indispensable, it is state-of-the-art to provide a web service for clinicians and research staff to track the status of data uploading on the cloud. The details of our data tracking website are provided in Figure 11 and Multimedia Appendix 1.

### Large Data Collected From the Pulsewatch System

In total, our Pulsewatch system collected 33,207,780 seconds (approximately 9224.38 h) and 182 GB of physiological recordings from the 90 participants who participated in phase 1 in the intervention group of 14 days and the 60 participants who participated in phase 2 (30 days).

For the functionality of the Pulsewatch system, as described in Figure 7, we recorded the raw PPG and ACC data for post hoc analysis because of the scarcity of smartwatch data sets for those with AF. The details of our output files from the Pulsewatch system are provided in the Multimedia Appendix 1.

As a staggering number of files were generated during the 44 days of continuous monitoring of the clinical trial, we had to consider the size of each file, as the cumulative size could be massive and pose challenges for the smartwatch, smartphone, and even the cloud server storage. The final file sizes were recorded during our clinical trial, and were found to be as large as 10 GB per participant if the Pulsewatch app was used daily. This amounts to a significantly large data storage requirement, considering that as many as 60 subjects could wear our Pulsewatch system for the entire study, lasting 44 days.

Because the quantity of data was quite substantial, we had to seek a large, long-term storage space on the cloud storage with a lower cost because the original storage space on the cloud server shown in Figure 6 is faster in read and write speed but costs much more. Details of the storage are described in Multimedia Appendix 1.

### Usability of the Pulsewatch System

Before phase 1 started in this clinical trial, we held a hack-a-thon meeting [14] to optimize the interactivity and usability of Pulsewatch, guided by information gleaned from 2 focus groups. From the hack-a-thon, the patients unanimously agreed that the UI layout shown above for both the phone and watch was clear and preferable to other options that were discussed.

After phase 1, researchers at UMass Chan studied the human factor aspects of our Pulsewatch system by surveying 90 patients who used it [25]. The participants used the System Usability Scale to quantify usability [25]. As documented in [25], patients (n=90) who received the Pulsewatch system had an average age of 65 years, 41% (37/90) were female individuals, and 87% (78/90) were of a White racial background. The baseline characteristics of the participants can be found in Table 1 of the study by Ding et al [25]. A total of 39% (32 /83) of patients found the system to be highly usable (System Usability Scale>68; [26]). For the watch app, 64% (56/88) of patients agreed or strongly agreed that it was easy to use. When considering only the phone app, 52% (46/88) of patients agreed or strongly agreed that it was easy to use. In addition, 42% (37/88) of patients felt that using Pulsewatch made them feel more connected to their clinicians. About one-eighth of the patients thought it was stressful to use the devices (11/88, 13% patients), but more than half enjoyed their experience using the system (45/88, 51% patients) [25].

### Formation of Smartwatch Wearing Habit

**Figure 12:**
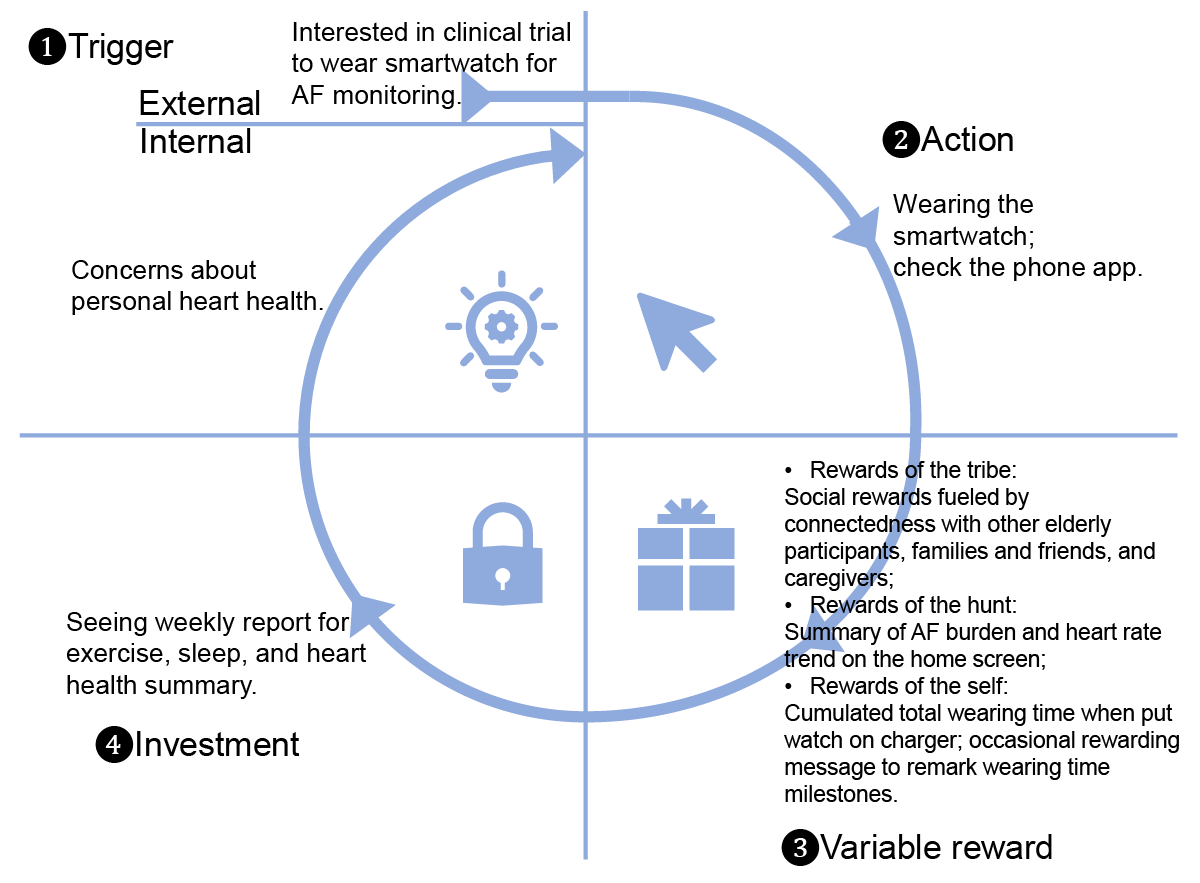
The Hook model forms the habit of using the Pulsewatch system. AF: atrial fibrillation.

On the first day of phase 2, 44 out of 57 (77%) participants in the intervention group wore the smartwatch of the Pulsewatch system [27]; however, it quickly dropped to only 14 out of 57 participants (25%) on the last day of phase 2. This fading enthusiasm for wearing a smartwatch for AF monitoring suggests that engineers and clinicians must consider the burden of the Pulsewatch system before designing its functionalities and user groups. In their book [28], Eyal described the “Hook” model to build user’s habit-forming products. In Figure 12, we illustrate how to use the Hook model to increase Pulsewatch system use among the targeted older adult population.

The Hook model consists of 4 steps to form the habit of using a new product. The first step is *trigger*, which means the actuator of a user’s behavior. An external trigger could be the health caregiver approaching the participant, and an internal trigger could be the participant’s concern for their health after stroke or TIA. We believe that all the enrolled participants experienced strong external and internal triggers in this study.

After the trigger comes the *action* step, which is defined as the user’s behavior in anticipation of a reward. The simple action in this study was to wear the Pulsewatch watch and sporadically check the AF results on the Pulsewatch phone app. This step could be challenging because the watch battery lasted only for 8 hours of recording. Consequently, participants may have lost patience with the frequent need to charge the watch. This situation will improve if developers have root access to the OS of smartwatches to shut down irrelevant smartwatch services for better battery management, and the battery capacity will increase as technology advances.

The third step is the *variable reward*, which is the key part that we believe should be improved upon. There are 3 variable types of rewards, and we believe that we can let end users select one or more types of rewards when they start to use the phone app. For rewards of the tribe, that is, when the users prefer to be supported by a community, they can see other participants’ wearing times and encourage other older adults to participate and adhere to the guidelines. If the caregivers or the users’ beloved families and friends are interested in helping the users, they can also join and provide in-app encouragement to the user. The momentum of wearing a watch can be continuously driven by social connections with others.

For those users who prefer rewards of the hunt, meaning they want information-intensive material, our phone app should provide a summary of AF burden and heart rate trends on the home screen that is easy for participants to find. Providing straightforward information reduces fatigue and encourages better adherence to data collection.

For users who prefer rewards of the self, that is, the need for intrinsic rewards of mastery, competence, and completion, one of the best ways to reward is to show the cumulative wearing time every time the user places the smartwatch on its charger. This is also helpful for participants with impaired cognitive function, as we could tailor some customized audio announcements to passively inform them of their accomplishments and health status. To increase the entertainment value of self-reward, we could provide occasional pop-up rewarding messages to remark on certain milestones of wearing time.

In the last step, which is the investment step, we can introduce a weekly report to the users summarizing their daily events, including exercise, sleep, heart health, and information on the time and effort the users have invested to leverage a new round of triggers.

## Discussion

### Principal Findings

As one of the first studies to provide a detailed design for a smartphone-smartwatch dyad for ambulatory AF monitoring, we successfully designed and implemented an innovative smartwatch-based AF monitoring mHealth solution for the older adult population with feedback from patients with AF and their clinicians. The usability of the system indicated an increase in the acceptability of mHealth solutions among older patients with cognitive impairment.

### Comparison With Prior Work

Although few manuscripts describe designs for health monitoring apps, Chae et al [29] described an approach to the design of a web-based upper limb home-based rehabilitation system using a smartwatch and smartphone for chronic stroke survivors. From the brief description of the system design by Chae et al [29], the recording of smartwatch data was initiated by touching the start button on the smartphone app, which required considerable attention from older adult patients. This was found to be difficult for the older adult participants to adhere to. Moreover, the frequency of rehabilitation exercises was as low as a few times a day; thus, the recording length was incomparable with the near-continuous recording of PPG data for AF monitoring in this study. Data processing was performed using the phone’s microprocessor, and not the watch, which would be an issue for real-time AF detection if Bluetooth is disconnected. Lutze et al [30-32] designed a stand-alone smartwatch app using the Samsung Simband smartwatch to handle health hazards for older people. However, other than simple tasks such as at-home checking through Wi-Fi connection, health reminders, and emergency calling, it neither performed other complicated tasks nor collected a large amount of data. Others have attempted to design combined smartphone and smartwatch apps as a diary self-management tool for diabetics [33]. The design of the app involved diabetic patients, but because the daily diary recording was discrete, the amount of data collected was minimal when compared with our AF monitoring; hence, cloud-based storage requirements were not needed. None of the above studies could provide a solution for freeing storage after recording a large amount of data, which is crucial for long-term monitoring, as storage space is limited in smartwatches (<8 GB).

### Limitations

The Pulsewatch system was designed to collect data for long-term monitoring, and the smartwatch file transfer process proceeded smoothly when the Bluetooth connection between the smartwatch and the smartphone was stable. However, several issues were encountered. For example, our longest recording streak among all participants was only 21 days during the combined 44 days of the phase 1 and phase 2 experiments. This was caused by improper functioning of the smartphone app during the 30-day phase 2. For example, the smartphone app could be terminated by the smartphone OS because of high battery consumption, or the participants may have their own smartphone device; therefore, they tend to forget to carry the study phone to maintain the Bluetooth connection of the Pulsewatch system. Consequently, the storage of the smartwatch became full, resulting in the loss of newly recorded information. In the future, smartwatch apps should have a data loss prevention mechanism when the watch storage is full. If the smartwatch app notices that the user has not maintained Bluetooth connectivity, and no files are uploaded to the smartphone for more than 3 or 4 days, it should automatically start a procedure to compress all existing files into a zip file, followed by deleting the original files to free up the data storage space. With this approach, we can save approximately 80% of the storage space, as the compression ratio for text files can be as high as 80% [34]. If this data compression functionality is implemented in the future, even without a Bluetooth connection to the phone, the Gear S3 smartwatch could record 24 days of data, and the Galaxy Watch 3 could record 64 days of data.

When we calculated the wearing time after the clinical trial was finished, we found that although obvious signs of wearing (eg, step counts) were observed in the Samsung Health smartwatch data, our Pulsewatch did not record any data. This could be caused by several factors, including full smartwatch storage. Although our watch app was tested to continuously and automatically operate on the watch, users could still terminate the running of our Pulsewatch app. Users can terminate our Pulsewatch app if they long press the watch face and switch to another built-in watch face. They could even accidentally delete our Pulsewatch app after long press the watch face. The Samsung Tizen Wearable OS has a battery use monitor, and it often asks users to stop running smartwatch apps that drain the battery. Furthermore, it automatically asks users to enter the *power saving* mode when the remaining battery is <20%. This power-saving mode is problematic because it terminates the operation of all third-party apps. Unless the watch is rebooted, our Pulsewatch app cannot automatically rerun again, even if users exit this mode after charging the watch. The Pulsewatch App must be active for rhythm detection, which has important implications for monitoring AF over an extended period.

In addition to wearing our Pulsewatch system, participants were also asked to wear the US Food and Drug Administration–approved, Cardea SOLO Wireless ECG Patch (Cardiac Insight). This served as the gold standard reference in phase 1 of the clinical trial to validate the accuracy of the AF monitoring algorithms for PPG. It should be noted that including a sample-level timestamp is crucial for any wearable system, especially on the reference device, as it is used to validate the accuracy of PPG data, such as peak detection comparison or heart rate variability analysis between the 2 devices. We added the sample-level timestamp for the Pulsewatch system after realizing that this capability was not enabled in the reference device when we enrolled 35 participants. Finally, we obtained the precise sample-level timestamp from the Cardiac Insight during the secondary analysis. Details of the sample-level timestamp issues are provided in Multimedia Appendix 1.

### Clinical Prospects

In this study, we detail potential issues in the design, development, and execution of a clinical trial that implements a novel digital health care system designed to monitor older stroke survivors for potential AF. The technical challenges encountered during the design and deployment process outlined in this study provide a foundational blueprint for future work in the area, both in research and clinical spaces, allowing for a more streamlined resource allocation and data management. In turn, this would lead to an improvement in patient outcomes. Our study also illustrated a successful and agile shift to internet-based recruitment in the context of the COVID-19 pandemic, providing a poignant example of how to adapt clinical trial protocols while maintaining data integrity and patient safety. Our study will enrich a diverse and inclusive pipeline of digital health and informatics professionals to address new pandemic-induced public health, medical, and scientific issues.

### Conclusions

As one of the first studies to provide a detailed design for a smartphone-smartwatch dyad for ambulatory AF monitoring, our team of engineers, programmers, clinicians, and patients successfully designed a system that has been used in a randomized clinical trial. The reported usability of our Pulsewatch system may increase the acceptability of mHealth solutions among older adult patients with cognitive impairment. Our proposed mHealth system overcomes some of the limitations of many prior devices for long-term AF monitoring. The Pulsewatch app was rated highly usable by over half of the stroke survivors in our study. All AF detection was performed solely on the smartwatch, and the smartphone served as a data transfer hub between the cloud and smartwatch. Clinicians organized the participant UIDs on the cloud and checked the collected data, including symptoms and notes logged by the participants on the Pulsewatch phone app. The Pulsewatch system successfully recorded raw data for subsequent data mining and machine-learning applications for AF detection.

## Appendix

Multimedia Appendix 1 Details of the implementation of the Pulsewatch system.

## Abbreviations

ACC: accelerometry
AF: atrial fibrillation
API: application programming interface
ECG: electrocardiogram
mHealth: mobile health
OS: operating system
pAF: paroxysmal atrial fibrillation
PPG: photoplethysmography
TIA: transient ischemic attack
UI: user interface
UID: user ID
UMass Chan: University of Massachusetts Chan Medical School

## Data Availability

The data collected from the Pulsewatch study are in the analysis phase; therefore, access to the data will be available once the study is completed. Once all data analyses have been completed, we will release the data to the public on our laboratory’s website [35]. The smartwatch and smartphone apps contain patented algorithms developed by the authors; therefore, they are not available on Google App stores or any open-source code repository.
