# Supplementary material for: A Smartwatch System for Continuous Monitoring of Atrial Fibrillation in Older Adults After Stroke or Transient Ischemic Attack: Application Design Study
Source: JMIR Cardio. 2023 Feb 13;7:e41691. doi: 10.2196/41691 (PMC9972205; doi:10.2196/41691)
Supplement: Multimedia Appendix 1 [file cardio_v7i1e41691_app1.docx]

# Multimedia Appendix 1. Details of the implementation of the Pulsewatch system.

### Results

### Implemented User Interface of the Smartwatch App

The heart rate value shown on the left side of the watch face reflects a 5-sec averaged heart rate from the Samsung heart rate API. The heart rate on the right side of the watch face represents an averaged heart rate from our real-time detection algorithm that was updated every 30 sec.

### Implementation of Smartphone App

The “Results” page allows a user to check a single AF detection record and log any symptoms or notes by clicking the view button after each record. A new screen with detailed information about heart rates and AF status pops up, and users could log symptoms or notes based on the record creation time.

The “My History” page provides a dashboard view to show the end-user’s heart rate trends for the past 24 hours, 7 days, or past 1 month. Filters are also provided to sift the displayed HR values for a certain HR range, certain time range, presence of AF, or any logged symptoms.

The last page was the “Get Help” page, where participants could find resources to learn more about AF and strokes. Due to COVID-19, some participants were enrolled entirely remotely, thus the “Get Help” page could provide video instructions with a playback function to inform the participants on how to apply the reference ECG patch for the Phase 1 participants and how to use the Pulsewatch system for all intervention group participants in both phases of the clinical trial.

### Output Files of the Pulsewatch System

During each ten minutes of the sensor-on stage, the watch app recorded 30 sec of sensor data, and saved them into a single file for that segment [1]. The smartwatch app generated ten 30-sec segments of PPG data with ten corresponding ACC data segments, one analytical log file, and one csv file every 10 minutes, as shown in Table 2. The sampling frequency for both PPG and ACC data was set at 50 Hz; the sampling frequency of 50 Hz for PPG signals has been shown to be sufficient for calculating features related to AF detection [2]. The sampling frequency for the ACC signal was set to be the same as that of the PPG signal so that the same data length could be obtained, and setting a sampling frequency for the ACC signal of at least 30 Hz was recommended for physical activity analysis [3]. A log file was also generated to document all of the signal processing output from the Pulsewatch algorithms, so that we could verify the arrhythmia detection accuracy for the post-hoc analysis. The log file also documented the software running status for any error debugging. A csv file was saved to record the AF status and heart rates that the users observed on the watch by the end of the sensor-on stage. In addition to all the standard data that each participant had access to, the customized symptoms and notes that were input by participants were saved by the web page in the phone app, thus they were synchronized directly inside the cloud server.

Table 2. Output files from the Pulsewatch system

| File Type (format) | Generated By | Content | Generated Frequency | File Size |
| --- | --- | --- | --- | --- |
| PPG files (*.txt) | Automatically by watch app | Column 1: timestamp in UNIX time;  Column 2: raw PPG data;  Column 3: Samsung Health heart rate value. | Every 30-sec during sensor-on stage (default 10 files per 10-min). | 103 KB if contains all 3 columns; 36 KB if only contains the 2^nd^ column (data column). |
| ACC files (*.txt) |  | Column 1: timestamp in UNIX time;  Column 2: raw ACC data. |  | 69 KB if contains all 2 columns; 36 KB if only contains the 2^nd^ column. |
| Analytical log files (*.txt) |  | Datetime timestamp, Software status and error message, 30-sec algorithm output (HR, motion artifact, and cardiac arrhythmia detection results). | When entering the sensor-off stage (default 1 file per 10-min). | 12 KB for default 5-min recording if contains timestamps for each row; 8 KB for default 5-min recording without any timestamp. |
| Watch displayed information file (*.csv) |  | UNIX timestamp, 5-sec averaged HR, 30-sec averaged HR, AF yes/no. | When entering the sensor-off stage (default 1 file per 10-min). | 1 KB |
| Symptoms and logs from users (*.csv) | Manually by end-users | Yes/no for each of the 6 listed symptoms; free text of notes. | If end-users entered any. | 1 KB |

The initial model of smartwatch was Gear S3 Classic (2016), which was equipped with a dual core 1.0 GHz adjunct processor, 768 MB RAM, and 4GB of internal memory [4]. After nearly a year of heavy use in the clinical trial, the battery health of the Gear S3s degraded significantly and could not support more than 3 hours of use, and this older model was discontinued as well. We replaced it with Galaxy Watch 3 (41mm) (2020) watch, which is equipped with a faster processor at 1.15 GHz, a larger RAM (1GB), and a larger internal memory of 8GB [5].

Based on the file size, shown in Table 2, a total of 1,733 KB of files would be generated every ten minutes. During 24 hours a day, 144 cycles of sensor-on stage usually occurred, so that the size of data generated per day was around 244 MB. The Gear S3 watch has 1.5 GB of free storage space for users while the Galaxy Watch 3 has more than 4 GB of free storage space. Therefore, the Gear S3 watch could collect about 6 days of data without unloading the data to the phone while the Galaxy Watch 3 could store ~16 days of data in the stand-by-mode.

### Implemented Data Tracking Website on the Cloud Server

This data tracking website served as the graphical user interface to allow the study admin to access three modules on the cloud server: the storage space of the standard data, such as AF results; the database of the customized data, which included user-reported symptoms and notes; and the database for user credential management, such as UIDs and passwords. The functionalities of the cloud web-service are shown in Fig. 11. The web admin could organize participant IDs and check uploaded data, including the symptoms and notes uploaded from the participants, on the secured cloud server. The UMass Chan team de-identified the participants and only provided UIDs to UConn team for patient confidentiality. On the server, organizing the uploaded files by UID folders and the date folders is extremely important, as the number of files being uploaded is extremely large. Moreover, a large list of file names could cause a long response time on the server when the phone app is requesting to upload new data, therefore, file uploading failure could happen even though everything appeared to be functioning correctly.

### Large Data Collected from the Pulsewatch System

Since the quantity of data was quite significant, we had to seek a large, long-term storage space on the cloud storage because the cloud server shown in Fig. 2 has only around 50 GB of storage. The fast read and write speed of the cloud server storage make the cost very expensive, resulting in limits to the space that we could use. Also, UConn IT Services only provides backup for executable image space, but not data storage space on the server, so if the server’s disks were damaged, we could lose the data. Eventually, a large amount of data was moved to a mapped UConn cloud drive for long-term storage, and it was backed up regularly by UConn ITS. The moving of data from the cloud server to a long-term drive was performed routinely by a UConn admin.

## Discussion

### Time-delay Due to Syncing Issue between the Reference ECG and the Smartwatch System

In addition to wearing our Pulsewatch system, participants were also asked to wear an FDA-approved ECG patch, Cardea SOLO Wireless ECG Patch (Cardiac Insight, Bellevue, WA, USA), as the gold standard reference in the Phase 1 of the clinical trial to validate the accuracy of the AF monitoring algorithms. We want to point out that including a sample-level timestamp is crucial for any wearable systems, especially on the reference device when it is used to validate the accuracy of another wearable device. Only when we started to align the reference ECG data with the corresponding watch PPG data after we enrolled 35 subjects in Phase 1, did we realize that a constant time-syncing issue existed either in the reference ECG patch or in the smartwatch system. We assumed that the provided timestamps were correct from Cardea SOLO raw ECG data as they showed a steady sampling frequency at 250 Hz, however, we spent significant amount of effort debugging the time accuracy of our Pulsewatch system. This is because we did not include an independent timestamp in the recorded data from Pulsewatch. This is why we added sample-level timestamps as individual columns in the PPG and ACC files in the midst of the clinical trial, as shown in Table 2, although we still had issues with aligning the Pulsewatch data with the reference ECG.


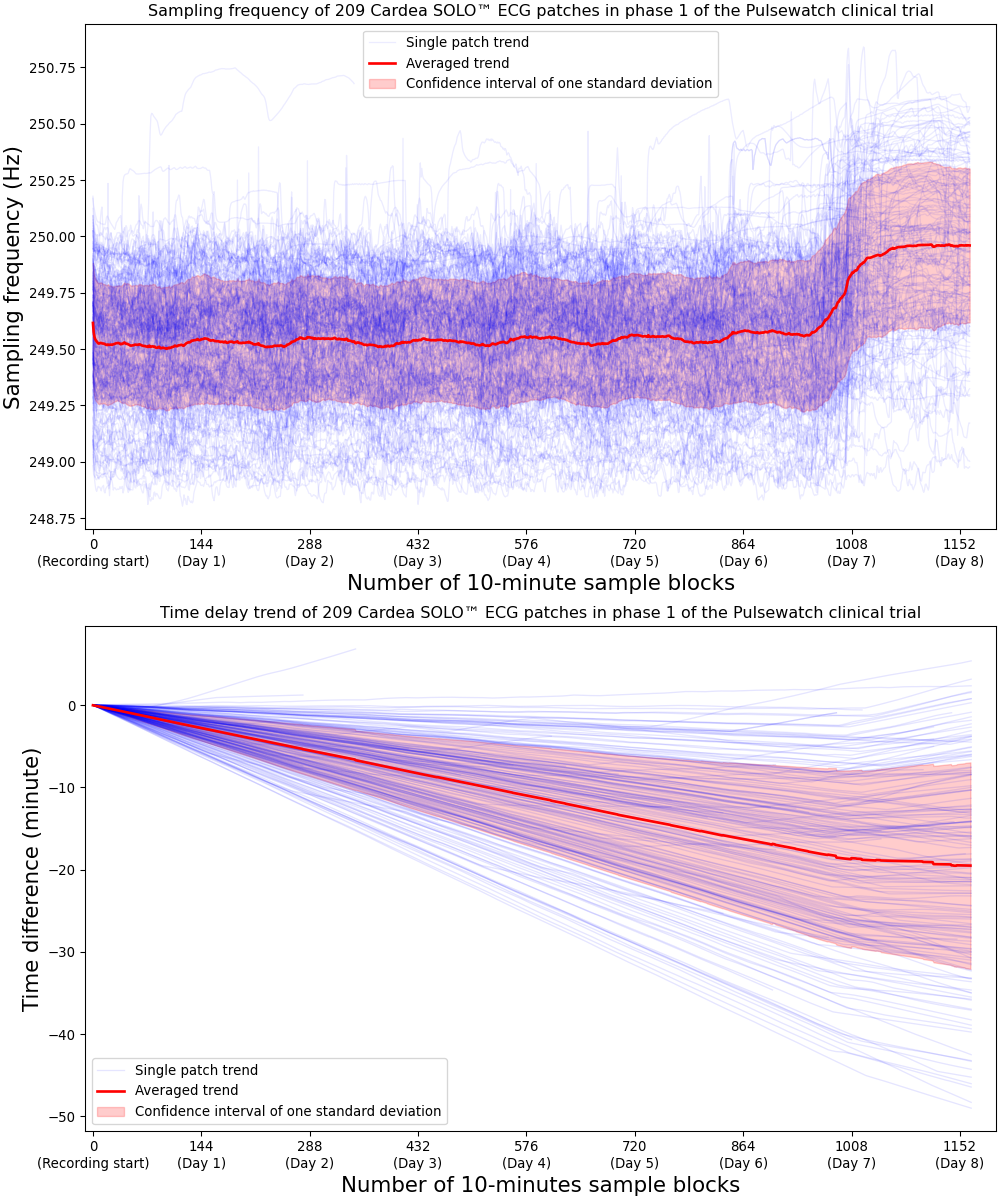


Figure 13. Top panel: illustrates fluctuations of sampling frequency for all 209 Cardea SOLO patches throughout the 7 days recording. Bottom panel: progressive time delay trends of all 209 Cardea SOLO patches throughout the 7 days recording. The x-axes in both panels represent the incremental of every 150k sample blocks Cardea SOLO recorded. In the top panel, the y-axis represents the actual sampling frequency for each sample block. In the bottom panel, the y-axis represents the cumulated time delay in the unit of 10-min sample blocks.

After the clinical trial ended, during the unblinding process of AF information inside the Cardea SOLO software, we learned that the misalignment problem was caused by the inconsistent sampling frequency of the ECG patch as it was fluctuating within a range of 250$\pm$2 Hz. To compound this complications, different ECG patches had different time syncing trends, which can be found in Fig. 13. According to Cardea Insight, when the sensor was first applied to the body, as the crystal inside the sensor warmed up, its properties changed leading to inconsistent sampling frequency from 249.5 Hz to 249.3 Hz (see Fig. 13). After a short while, the sample rate became more stable and slowly varied around 249.3 Hz, as shown in Fig. 13. Although 249.3 Hz versus 250 Hz is not a large difference, if we have 151.2 million samples recorded for a 7-day recording, the actual time difference accumulated between two different sampling frequencies could be as large as 28.3 minutes.

Subsequently, with the additional software Cardea Insight provided, we received the “precise” timing from ECG, but the resolution was still not in a sample-level but only in every 150k sample block. For example, the precise timing was recorded in the patch for each increment of 150,000 samples (equivalent to 10 minutes if sampling frequency at exactly 250 Hz). In collaboration with Cardiac Insight, we performed linear interpolation within each 10-min time block to fetch a sample-level timestamp. This could lead to potential misalignment within each 10-min since the fluctuation of sampling frequency was non-linear within each 10-min period as shown in Fig. 13. We are sharing this information to suggest that developers seeking to compare data between ECG and wearable devices to add the sample-level timestamp for any recorded signal in the case precise data analysis is needed,

## Reference

[1] J. S. Steinberg, H. O’Connell, S. Li, and P. D. Ziegler, “Thirty-Second Gold Standard Definition of Atrial Fibrillation and Its Relationship With Subsequent Arrhythmia Patterns,” *Circulation: Arrhythmia and Electrophysiology*, vol. 11, no. 7, p. e006274, Jul. 2018, doi: 10.1161/CIRCEP.118.006274.

[2] S. Béres and L. Hejjel, “The minimal sampling frequency of the photoplethysmogram for accurate pulse rate variability parameters in healthy volunteers,” *Biomedical Signal Processing and Control*, vol. 68, p. 102589, Jul. 2021, doi: 10.1016/j.bspc.2021.102589.

[3] J. H. Migueles *et al.*, “Accelerometer Data Collection and Processing Criteria to Assess Physical Activity and Other Outcomes: A Systematic Review and Practical Considerations,” *Sports Med*, vol. 47, no. 9, pp. 1821–1845, Sep. 2017, doi: 10.1007/s40279-017-0716-0.

[4] “Samsung Gear S3 classic,” *Samsung Mobile Press | Samsung Mobile press Official website*. https://www.samsungmobilepress.com/mediaresources/gear-s3-classic/techspecs (accessed Jan. 20, 2022).

[5] “Specs | Samsung Galaxy Watch3,” *The Official Samsung Galaxy Site*. https://www.samsung.com/global/galaxy/galaxy-watch3/specs/ (accessed Jan. 20, 2022).
